# Progress in the Development of CdTe and CdZnTe Semiconductor Radiation Detectors for Astrophysical and Medical Applications

**DOI:** 10.3390/s90503491

**Published:** 2009-05-12

**Authors:** Stefano Del Sordo, Leonardo Abbene, Ezio Caroli, Anna Maria Mancini, Andrea Zappettini, Pietro Ubertini

**Affiliations:** 1 INAF/IASF Palermo, Via Ugo La Malfa 153, 90146 Palermo, Italy; 2 Dipartimento di Fisica e Tecnologie Relative, Università di Palermo,Viale delle Scienze, 90128 Palermo, Italy; 3 INAF/IASF Bologna, Via Gobetti 101, 40129 Bologna, Italy; E-Mail: ezio.caroli@iasfbo.inaf.it (E.C.); 4 Dipartimento di Ingegneria dell'Innovazione, Università del Salento, Via Arnesano, 73100 Lecce, Italy; E-Mail: annamaria.mancini@unile.it (A.M.M.); 5 IMEM-CNR, Parco Area delle Scienze 37/A, 43100 Parma, Italy; E-Mail: zapp@imem.cnr.it (A.Z.); 6 INAF/IASF Roma, Via del Fosso del Cavaliere 100, 00133 Roma, Italy; E-Mail: pietro.ubertini@iasf-roma.inaf.it (P.U.)

**Keywords:** compound semiconductors, CdTe and CdZnTe detectors, X-ray and gamma ray spectroscopy

## Abstract

Over the last decade, cadmium telluride (CdTe) and cadmium zinc telluride (CdZnTe) wide band gap semiconductors have attracted increasing interest as X-ray and gamma ray detectors. Among the traditional high performance spectrometers based on silicon (Si) and germanium (Ge), CdTe and CdZnTe detectors show high detection efficiency and good room temperature performance and are well suited for the development of compact and reliable detection systems. In this paper, we review the current status of research in the development of CdTe and CdZnTe detectors by a comprehensive survey on the material properties, the device characteristics, the different techniques for improving the overall detector performance and some major applications. Astrophysical and medical applications are discussed, pointing out the ongoing Italian research activities on the development of these detectors.

## 1. Introduction

Semiconductor nuclear radiation detectors have experienced a rather rapid development in the last few years. They are now used in a large variety of fields, including nuclear physics, X-ray and gamma ray astronomy and nuclear medicine. Their imaging capabilities, good energy resolution and the ability to fabricate compact systems are very attractive features, in comparison with other types of detectors, such as gas detectors and scintillators. In recent years, a substantial international effort has been invested in developing a range of compound semiconductors with wide band gap and high atomic number for X-ray and gamma ray detectors. Among the compound semiconductors, cadmium telluride (CdTe) and cadmium zinc telluride (CdZnTe) are the most promising materials for radiation detectors with good energy resolution, high detection efficiency and room temperature operation.

This paper is a review of recent advances in the development of CdTe and CdZnTe radiation detectors. The main properties of compound semiconductor detectors, the characteristics of CdTe and CdZnTe detectors, the different techniques for improving the overall detector performance and some major applications, will be presented and discussed. International and, especially, Italian activities on the development of CdTe and CdZnTe detectors for medical and astrophysical applications will be also reviewed.

## 2. Room-Temperature Compound Semiconductor Radiation Detectors

Silicon (Si) and germanium (Ge) are traditional semiconductors used for radiation detectors that offer good performance in a wide range of applications [1]. The growing field of applications has stimulated the development of detectors based on compound semiconductors [1-3]. They were first investigated as radiation detectors in 1945 by Van Heerden [4], who used AgCl crystals for detection of alpha particles and gamma rays. A great advantage of compound semiconductors is the possibility to grow materials with a wide range of physical properties (band gap, atomic number, density) making them suitable to almost any application. Interests in radiation detectors operating at room temperature gave rise to development of compound semiconductors with wide band gaps, in comparison to Si and Ge. Moreover, for X-ray and gamma ray detection, compound semiconductors with high atomic number were preferred in order to emphasize photoelectric interaction. Compound semiconductors are generally derived from elements of groups III and V (e.g. GaAs) and groups II and VI (e.g. CdTe) of the periodic table. Besides binary compounds, ternary materials have been also produced, e.g. CdZnTe and CdMnTe. Table 1 reports the physical properties of the most common compound semiconductors typically used for radiation detection.

Among the compound semiconductors, CdTe and CdZnTe have attracted growing interests in the development of X-ray and gamma ray detectors [5,6]. Due to the high atomic number, the high density and the wide band gap, CdTe and CdZnTe detectors ensure high detection efficiency, good room temperature performance and are very attractive for X-ray and gamma ray applications.

Difficulties in producing detector-grade materials and in growing chemically pure and structurally perfect crystals are the critical issues of CdTe and CdZnTe detectors. In fact, the great potential of these compounds has not been exploited for many decades due mainly to the limited commercial availability of high-quality crystals. This situation has changed dramatically during the mid-nineties with the emergence of a few companies committed to the advancement and commercialization of these materials.

### 2.1. X-ray and gamma ray detection with semiconductors: principles of operation

**Figure 1.**
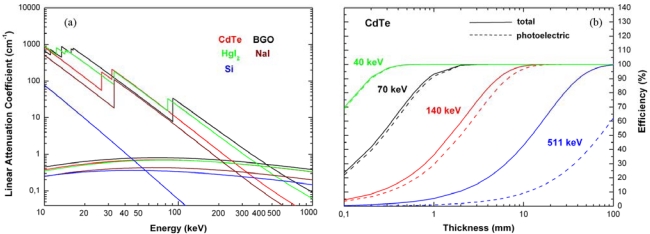
(a) Linear attenuation coefficients for photoelectric absorption and Compton scattering of CdTe, Si, HgI_2_, NaI and BGO. (b) Efficiency of CdTe detectors as function of detector thickness at various photon energies.

The typical operation of semiconductor detectors is based on collection of the charges, created by photon interactions, through the application of an external electric field. The choice of the proper semiconductor material for a radiation detector is mainly influenced by the energy range of interest. Among the various interaction mechanisms of X-rays and gamma rays with matter, three effects play an important role in radiation measurements: photoelectric absorption, Compton scattering and pair production. In photoelectric absorption the photon transfers all its energy to an atomic electron, while a photon interacting through Compton process transfers only a fraction of its energy to an outer electron, producing a hot electron and a degraded photon; in pair production a photon with energy above a threshold energy of 1.02 MeV interacts within the Coulomb field of the nucleus producing an electron and positron pair. Neglecting the escape of characteristic X-rays from the detector volume (the so called fluorescent lines), only the photoelectric effect results in the total absorption of the incident energy and thus gives useful information about the photon energy. The interaction cross sections are highly dependent on the atomic number. In photoelectric absorption it varies as Z^4,5^, Z for Compton scattering and Z^2^ for pair production. An optimum spectroscopic detector must favor photoelectric interactions and so semiconductor materials with a high atomic number are preferred. Figure 1(a) shows the linear attenuation coefficients, calculated by using tabulated interaction cross section values [7], for photoelectric absorption and Compton scattering of Si, CdTe, HgI_2_, NaI and BGO; NaI and BGO are solid scintillator materials typically used in radiation measurements. As shown in Figure 1(a), photoelectric absorption is the main process up to about 200 keV for CdTe. The efficiency for CdTe detectors versus detector thickness and at various typical photon energies is reported in Figure 1(b). A 10 mm thick CdTe detector ensures good photoelectric efficiency at 140 keV (> 90%), while a 1 mm thick CdTe detector is characterized by a photoelectric efficiency of 100% at 40 keV.

**Figure 2.**
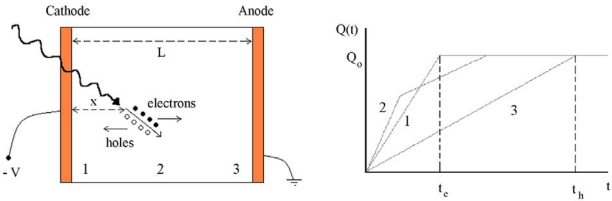
Planar configuration of a semiconductor detector (left). Electron–hole pairs, generated by radiation, are swept towards the appropriate electrode by the electric field. (right) The time dependence of the induced charge for three different interaction sites in the detector (positions 1, 2 and 3). The fast rising part is due to the electron component, while the slower component is due to the holes.

Semiconductor detectors for X-ray and gamma ray spectroscopy behave like solid-state ionization chambers operated in pulse mode. The simplest configuration is a planar detector, i.e. a slab of a semiconductor material with metal electrodes on the opposite faces of the semiconductor (Figure 2). Photon interactions produce electron-hole pairs in the semiconductor volume through the discussed interactions. The interaction is a two-step process where the electrons created in the photoelectric or Compton process lose their energy through electron-hole ionization. The most important feature of the photoelectric absorption is that the number of electron-hole pairs is proportional to the photon energy. If *E* is the incident photon energy, the number of electron-hole pairs *N* is equal to *E/w*, where *w* is the average pair creation energy. The generated charge cloud is *Q_0_= eE/w*. The electrons and holes move toward the opposite electrodes, anode and cathode for electrons and holes, respectively (Figure 2). The movement of the electrons and holes, causes a variation ΔQ of induced charge on the electrodes. It is possible to calculate the induced charge ΔQ by the Shockley-Ramo theorem [8-11] which makes use of the concept of the weighting potential *φ*. The weighting potential is defined as the potential that would exist in the detector with the collecting electrode held at unit potential, while holding all other electrodes at zero potential. According to the Shockley–Ramo theorem, the induced charge by a carrier *q*, moving from *x_i_* to *x_f_*, is given by:

(1) $$\Delta Q=-q\left[\varphi (x_{f})-\varphi (x_{i})\right]$$

where *φ* (x) is weighting potential at position *x*. It is possible to calculate the weighting potential by analytically solving the Laplace equation inside a detector [12]. In a semiconductor, the total induced charge is given by the sum of the induced charges due both to the electrons and holes. For a planar detector, the weighting potential *φ* of the anode is a linear function of the distance *x* from the cathode:

(2) $$\begin{array}{cc} \varphi \left(x\right)=\frac{x}{L} & \;0\leq \frac{\text{x}}{\text{L}}\leq 1 \end{array}$$

where *L* is the detector thickness. Neglecting charge loss during the transit time of the carriers, the charge induced on the anode electrode by *N* electron-hole pairs is given by:

(3) $$\begin{array}{c} \Delta Q=\Delta Q_{h}+\Delta Q_{e}=-\frac{\left(\text{Ne}\right)}{L}(0-x)+\frac{\left(\text{Ne}\right)}{L}(L-x)=Ne=Q_{0};\\ t>t_{h}=\frac{x}{\mu_{h}E}t>t_{e}=\frac{L-x}{\mu_{e}E} \end{array}$$

where *t_h_* and *t_e_* are the transit times of holes and electrons, respectively.

**Figure 3.**
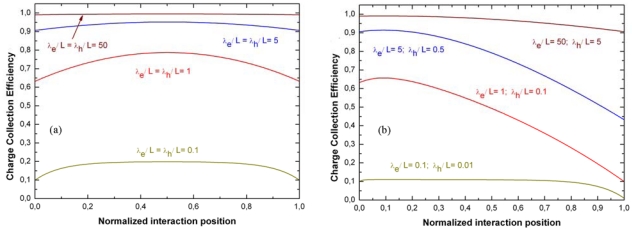
Charge collection efficiency (CCE) vs. the normalized interaction position of incoming photons. The CCE curves are calculated for different values of the λ/L ratios; (a) identical λ/L ratios for both electrons and holes; (b) the electron λ/L ratio is always 10 times greater than that of the holes.

Charge trapping and recombination are typical effects in compound semiconductors and may prevent the full charge collection. For a planar detector, having a uniform electric field, neglecting charge de-trapping, the charge collection efficiency (CCE), i.e. the induced charge normalized to the generated total charge, is given by the Hecht equation [13]:

(4) $$\text{CCE}=\frac{Q}{Q_{0}}=\left[\frac{\lambda_{h}}{L}\left(1-e^{-\frac{x}{\lambda_{h}}}\right)+\frac{\lambda_{e}}{L}\left(1-e^{-\frac{L-x}{\lambda_{e}}}\right)\right]$$

where *λ_h_= μ_h_τ_h_E* and λ_e_ = *μ_e_τ_e_E* are the mean drift lengths of holes and electrons, respectively. The CCE depends not only on *λ_h_* and λ_e_, but also on the incoming photon interaction position. The random distribution of the interaction point increases the fluctuations on the induced charge and thus produces peak broadening in the energy spectrum. Small λ/L ratios reduce the CCE and increase the dependence by the interaction point, as shown in Figure 3.

The charge transport properties of a semiconductor, expressed by the hole and electron mobility lifetime products (*μ_h_τ_h_* and *μ_e_τ_e_*) are key parameters in the development of radiation detectors. Poor mobility lifetime products result in short *λ* and therefore small *λ /L* ratios, which limit the maximum thickness and energy range of the detectors. Compound semiconductors, generally, are characterized by poor charge transport properties due to charge trapping. Trapping centers are mainly caused by structural defects (e.g. vacancies), impurities and irregularities (e.g. dislocations, inclusions). In compound semiconductors, the *μ_e_τ_e_* is typically of the order of 10^-5^-10^-3^ cm^2^/V while *μ_h_τ_h_* is usually much worse with values around 10^-6^-10^-4^ cm^2^/V, as reported in Table 1. Therefore, the corresponding mean drift lengths of electrons and holes are 0.2-20 mm and 0.02-2 mm, respectively, for typical applied electric fields of 2,000 V/cm.

The charge collection efficiency is a crucial property of a radiation detector and affects the spectroscopic performance and in particular the energy resolution. High charge collection efficiency ensures good energy resolution which also depends by the statistics of the charge generation and by the noise of the readout electronics. Therefore, the energy resolution (FWHM) of a radiation detector is mainly influenced by three terms:

(5) $$\Delta E=\sqrt{{\left(2.355\right)}^{2}(F\cdot E\cdot w)+\Delta {E_{\text{el}}}^{2}+{\Delta E_{\text{coll}}}^{2}}$$

The first term is the Fano noise due to the statistics of the charge carrier generation. In semiconductors, the Fano factor *F* is much smaller than unity (0.06 – 0.14) [14]. The second term is the electronic noise, which is generally measured directly using a precision pulser, while the third is the contribution of the charge collection process. Several semi-empirical relations have been proposed for the charge collection term of different detectors [3,15].

**Figure 4.**
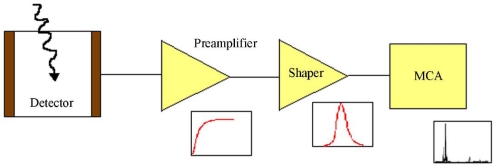
Block diagram of a typical spectroscopy system.

Figure 4 shows a typical spectroscopic system based on a semiconductor detector. The detector signals are amplified by a charge sensitive preamplifier (CSP) and then shaped by a linear amplifier (shaping amplifier). Energy spectra are obtained by a multichannel analyzer (MCA) which samples and records the shaped signals.

## 3. CdTe Detectors

Cadmium telluride has been studied as an X-ray and gamma ray detector material since the 1960s [16]. CdTe has a cubic zincblende crystal structure with atomic numbers of 48 (Cd) and 52 (Te) and a wide band gap of 1.44 eV, which guarantees room temperature operation. Poor charge carrier transport properties and disparity between electrons and holes properties are typical of CdTe materials (*μ_e_τ_e_* = 10^-3^ cm^2^/V and *μ_h_τ_h_* = 10^-4^ cm^2^/V). The low values of the charge carrier mobility-lifetime products are due to the presence of defects and impurities in the crystals which act as trapping centres. Typical defects in CdTe are mainly structural defects, impurities and complexes of the two, e.g. cadmium vacancies and donor impurities (Cl, In) [17]. The principal physical properties of CdTe are reported in Table 1. High purity CdTe crystals can be grown by using different techniques, such as zone melting, the Bridgman method, epitaxial and travelling heater method (THM) [17]. CdTe crystals are usually grown by the THM method and doped with Cl to compensate background impurities and defects, resulting in high resistivity *p*- type materials (10^9^ Ω cm). Supplies of spectrometer grade CdTe crystals are offered by a few companies: Imarad (Israel), Eurorad (France) and Acrorad (Japan). CdTe detectors are generally fabricated by using metal-semiconductor-metal (MSM) structures. Metals with a high work function, such as gold and platinum, form ohmic contacts while diode behavior (blocking contacts) is obtained with In contacts. A critical issue of CdTe detectors is their time instability under bias, i.e. the so-called polarization effect [18,19]. The term polarization has come to mean any change in the performance of the detectors after the detector biasing. This effect is mainly due to the trapping and the de-trapping of the carriers that affect the space-charge distribution and the electric field profile in the detectors. Polarization leads to a time-dependent decrease in counting rate and charge collection efficiency. It is possible to minimize the polarization effects by using high bias voltages and low temperature operation [19]. CdTe detectors with blocking contacts and cooled with compact Peltier cells showed good performance and time stability [19]; an energy resolution of 3% (FWHM) at 59.5 keV (T = 10 °C) characterized the ^241^Am spectra measured with a 1 mm thick CdTe detector with blocking contacts [19].

In the following paragraph we describe, in detail, the epitaxial growth method of CdTe. In particular, we report on the experimental activities of an Italian research group involved on the development of CdTe detectors based on epitaxially grown crystals [20].

### 3.1. Epitaxial growth technology for CdTe detectors

Since the last decade some epitaxial growth technology for CdTe detectors are developing. In particular two research lines are evolving: thick or thin film deposition. *Thick film deposition* is used in order to optimize the electrical properties of CdTe crystals and to minimize large inhomogeneities in the crystals.

Also, good quality CdTe single crystals achieved by melt-growth technologies are limited, in industrial production, to less than 1.5 in. diameter in size by severe structural problems. Instead, vapour-phase crystallisation of CdTe has several advantages over melt-growth, such as lower growth temperatures (which allow the reduction of native point defect concentrations), higher material purity, careful control of the VI/II stoichiometry, and the ease by which the compensating dopants can be introduced into the vapour.

Although many of such advantages are common to both bulk and epitaxial vapour growth methods, most efforts have been focussed on the vapour growth of bulk CdTe, whilst vapour-phase epitaxy (VPE) methods for the realisation of detector-grade CdTe have not yet been explored. Of course, the realisation of X-ray and gamma ray detectors for photon energies above 100 keV requires several mm of material which can only be achieved by bulk growth methods. However, X-ray detection in the 1-50 keV photon energy range requires sensitive material corresponding to less than 500 micron thick CdTe crystals. This brings the growth of the detector-grade CdTe within the limits of VPE methods; these would have the additional advantage of allowing CdTe growth on large (2 in.) areas with a high degree of lateral homogeneity.

**Figure 5.**
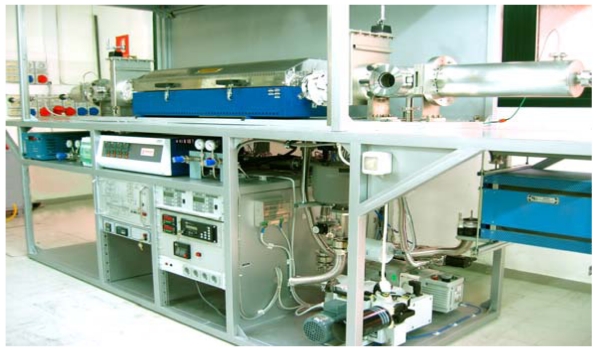
H_2_ transport – vapour phase epitaxy reactor.

**Figure 6.**
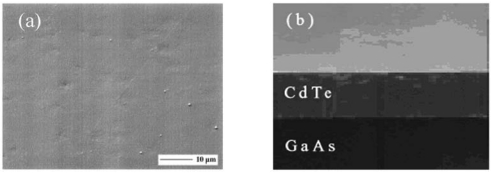
SEM micrographs of (a) surface plan view and (b) cleaved cross section of a CdTe epilayer on GaAs substrate.

The growth of thick CdTe epitaxial layers by the hydrogen transport vapor phase epitaxy (H_2_T-VPE) method has been reported [20-22]. The thermodynamics of the H_2_ transport method of CdTe were analyzed to determine the equilibrium partial pressures of the molecular species in the vapor and its supersaturation as a function of growth conditions. (100)-oriented CdTe epilayers are successfully grown by H_2_T-VPE on hybrid ZnTe/GaAs(100) substrates prepared by metalorganic vapor phase epitaxy. Growth rates up to 10 μm/h are obtained at temperatures ∼ 760 °C and with the CdTe source temperature at 827 °C. The reactor, on purpose realised, is shown in Figure 5. The achievement of even higher growth rates can be foreseen by using the present method under slightly different conditions; several hundreds micron thick CdTe layers can be thus grown by the H_2_T-VPE. CdTe samples have mirror-like, nearly featureless surfaces as shown in Figure 6. After epilayer growth, the underlying GaAs substrate is completely removed by chemo-mechanical thinning and etching until a free-standing CdTe thick crystal slab is obtained. Also, CdTe epilayers have shown a medium-to-high resistivity at room temperature, possibly as a result of the compensation by donor impurities diffusing from GaAs. Still the growth of highly resistive layers by *in-situ* chlorine doping during the H_2_T-VPE growth is possible. In summary, H_2_T-VPE is a potential alternative to traditional melt- and vapor-growth methods for the synthesis of detector-grade CdTe for application to the 1 – 50 keV x-ray energy range.

The *Thin film deposition* technique originates from the consideration that, at present, CdTe-based detectors employ In or Pt thin films to form a Schottky barrier (blocking contact) or an ohmic contact, respectively, onto semi-insulating CdTe crystals; however, the device fabrication presents numerous problems: (i) it is very difficult to fabricate metal contacts onto seminsulating CdTe crystals with reproducible electrical characteristics due to the extreme sensitivity of their electrical properties to fabrication conditions and (ii) the need to apply high bias voltages to the device to achieve good charge collection efficiency leads to relatively high dark current values (10^−7^ – 10^−8^ A/cm^2^).

**Figure 7.**
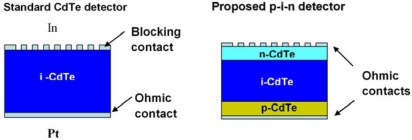
Standard and proposed detector structures.

Therefore a new structure has been proposed: CdTe detectors based on homoepitaxial p–i–n diode structures, in which the intrinsic part is a high-resistivity (detector-grade) bulk crystal, while the n- and p-doped ones are homoepitaxial layers, should be able to show much lower values of the dark current. The two typologies are compared in Figure 7. However, the performance of p-i-n diode detectors strongly depend on the quality of the homoepitaxial p- and n-type contacts onto detector-grade CdTe crystals, whose deposition and post-growth processing thus represent critical fabrication steps. In particular, while p-type doping of CdTe is relatively straightforward, the growth of highly conducting n-type layers appears more difficult.

**Figure 8.**
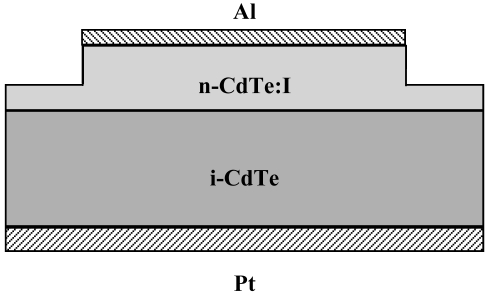
Schematic of the Al/n-CdTe:I/i-CdTe/Pt structure. The n-CdTe:I layer below the Al electrode is 2 μm thick.

Although the epitaxy of CdTe on (111)-oriented CdTe crystals was the subject of many reports around two decades ago [23,24], most of those studies were focused on the epitaxy of HgCdTe-related materials for infrared device applications. Until very recently no data were presented in the literature concerning the homoepitaxy of CdTe on detector-grade crystals. The growth of I-doped CdTe on high resistivity (111)B crystals by remote-plasma-enhanced metalorganic vapour phase epitaxy (MOVPE) was first reported in 2002 [25]. However, no detailed information on substrate surface treatments before growth and their effects on the crystalline quality of the CdTe layers was presented by the authors. The application of Metal Organic Vapour Phase (MOVPE) technology to the homoepitaxy of n-CdTe:I layers on commercial detector-grade CdTe crystals is reported in [26-28]. This is a preliminary technological step towards the fabrication of CdTe-based p-i-n diode X/gamma-ray detectors. The realized structure is shown in Figure 8. Dimethylcadmium (Me_2_Cd), di-isopropyl-telluride (iPr_2_Te) and ethyl iodide (EtI), were used as Cd, Te and I precursors, respectively. Detector-grade CdTe crystals with resistivity > 10^8^ Ω·cm were supplied by Acrorad, Ltd. (Japan). They were nominally (111)-oriented, 1 × 1 cm^2^ large and 1 mm thick.

In order to achieve good epitaxy, as-received substrates had to be carefully prepared before growth by etching in Br_2_-methanol and subsequently treated in the MOVPE reactor by H_2_ heat-cleaning at 350 °C. An additional annealing at 350 °C under a H_2_ + Me_2_Cd vapor was afterwards applied to the wafers for 5 min, as this treatment proved to ensure the reproducible single crystalline growth of the overgrown epilayer. Homoepitaxial (111)-oriented layers with fairly good surface morphology were obtained at 330 °C on as-prepared substrates by growing under Cd-rich vapour stoichiometry conditions. Typical material growth rates were around 2.6 μm/h and 2.0 μm/h under stoichiometric and Cd-rich vapor conditions, respectively. Sample thickness ranged in the 2.0 – 4.0 μm interval.

**Figure 9.**
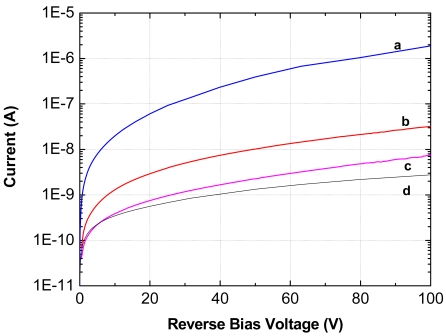
Comparison between standard and new structures leakage currents: (a) Pt/CdTe/Pt commercial device; (b) i-n detector before H_2_O_2_ treatment, (c) i-n detector after H_2_O_2_ treatment and d) In/CdTe/Pt commercial device.

Doping of the layers with I during MOVPE growth allowed to achieve n-type conductivity with CdTe resistivity values of a few Ω cm and carrier concentrations limited to ∼ 10^16^ cm^-3^. Electrical and photoluminescence (PL) measurements suggest a substantial degree of donor electrical compensation, likely by unintentional formation of V_Cd_-I_Te_ acceptor centres in the material. The compensation mechanism is described in reference [29]. The I-V characteristics of preliminary M-i-n structures fabricated from present n-CdTe:I/i-CdTe structures evidence a reduced leakage current paths in the device among a traditional Pt/Pt detector. Moreover the passivation of the device surface further improve the overall I-V characteristics of the M-*i*-n structures. Figure 9 [30] shows the effectiveness of H_2_O_2_ treatment for the suppressing parasitic currents and that i-n detector leakage current is almost 3 orders of magnitude lower than Pt/Pt device, while In/Pt device leakage current is lowest, but its polarization phenomena are well known (polarization phenomena are due to the de-trapping of holes from deep acceptors that creates a negative space charge accumulation at the In anode of the detectors [18,19,31]). Leakage currents can be further suppressed in the final p-i-n structure.

More work is in progress to achieve higher electron concentrations in the n-type CdTe layer, as well as the growth of p-type material for the realization of the final p-i-n diode structure.

## 4. CdZnTe Detectors

Cd_1-x_Zn_x_Te is a more recent candidate for room temperature radiation detectors [32,33]. The addition of a few percent of zinc to the melt results in an increased band gap as well as the energy of defect formation. CdTe and ZnTe form a solid solution throughout the entire alloy range, however only the range x = 0.1 – 0.2 is used for detector applications (x is the blending fraction of ZnTe in CdTe). The increased band gap (1.57 eV, x = 0.1) ensures high bulk resistivities and reduces the dislocation density, resulting in lower leakage currents and higher temperature operation. Specifically, resistivities of CdZnTe are typically between one and two orders of magnitude greater than that of CdTe and thus leakage currents are correspondingly lower. This is important in the detector development, because allows to obtain a lower leakage current or to apply a larger voltage bias. This ternary compound has a cubic, zincblende-type lattice with atomic number close to that of CdTe. The main drawback of CdZnTe crystals is the low value of μτ of the carriers and especially the major difference between the μτ values of electrons and holes in respect to CdTe (Table 1). However, the main advantage of CdZnTe over CdTe detectors is the absence of the polarization effect, that limits the exploitation of CdTe detectors.

CdZnTe crystals are usually grown by using the high pressure Bridgman (HPB), low pressure Bridgman (LPB), vertical Bridgman and THM methods. The supply of spectrometer grade CdZnTe is limited to a small number of companies: eV Products (USA), Imarad (Israel), Eurorad (France) and Redlen Technologies (Canada). Due to their low leakage currents (< 10 nA at room temperature), CdZnTe detectors are usually fabricated with ohmic contacts (Pt, Au) by using metal-semiconductor-metal (MSM) structures. CdZnTe detectors with ohmic contacts (Pt) showed good energy resolution of 1.4% (FWHM) at 59.5 keV (T = -37 °C) [3]. Nevertheless, the poor hole charge transport properties produce long tails in the measured spectra. In order to overcome this problem single carrier devices, i.e. electron sensitive detectors, have been developed, as it will be discussed later.

In spite of the research efforts to improve the crystal growth technology, the yield of detector quality material remains low. This is because the growth of CdZnTe crystals presents some intrinsic difficulties: i) the superheating required for eliminating polymer in the melt above the melting point that makes seeding very difficult, ii) the low thermal conductivity of the solid, iii) the not-congruent evaporation of CdZnTe at the melting point, iv) the tendency of the matrix to incorporate tellurium inclusions, v) the low value of the critical resolved shear stress (CRSS) that facilitates the formation of a large number of dislocations. Among these, the superheating required for eliminating polymer in the melt above the melting point that makes seeding very difficult, the low thermal conductivity of the solid, and the not-congruent evaporation of CdZnTe at the melting point. In this frame, some of the authors developed a new technique for the growth of CdZnTe crystals that is known as boron oxide vertical Bridgman technique (section 4.1).

In parallel to the main activity on the tuning of grown technique to produce CdZnTe crystals with the required performance (high resistivity, good charge collection efficiency, etc.), several efforts have been also dedicated on the development of a reliable method to metalize the surface for the realization of efficient contacts. Planar detectors have been prepared out from these crystals using electroless technique to realize Au metallic mono-electrode on the opposite surfaces in order to evaluate the spectroscopic performance of sensors made of the grown material (section 4.2).

### 4.1. CdZnTe crystals grown by the boron oxide encapsulated vertical Bridgman technique

One of the problems limiting the widespread exploitation of CdZnTe based devices is the low single crystal yield that still affects the crystal growth processes. One of the reasons for the formation of twins and grain boundaries is the crystal-crucible interaction. Particularly detrimental seems to be the use of quartz crucibles that ease the sticking of the crystal to the crucible walls. Due to this reason, many authors suggested the use of graphite crucibles [34], carbon coated quartz crucibles [35] or pBN crucibles [36].

In principle, it is possible to avoid the contact between the growing crystal and the crucible in a vertical Bridgman configuration, by opportunely controlling the crucible/melt/crystal wetting angles [37]. However, it was shown that, in the case of crystals like Ge, CdTe and GaSb, this is possible only under microgravity conditions [38] or by imposing a pressure difference between the melt and the solidifying crystal [39,40]. While the former solution is of course not applicable to production, the latter suffers many technical difficulties.

**Figure 10.**
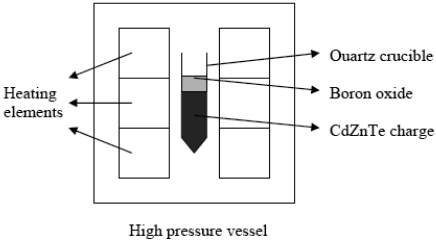
Growth set up of the boron oxide encapsulated vertical Bridgman technique.

In order to overcome the problem of the crystal-crucible interaction, an Italian group working at IMEM-CNR has developed a novel growth technique, called Boron Oxide Encapsulated Vertical Bridgman Technique [41-43]. The growth set up is shown in Figure 10.

**Figure 11.**
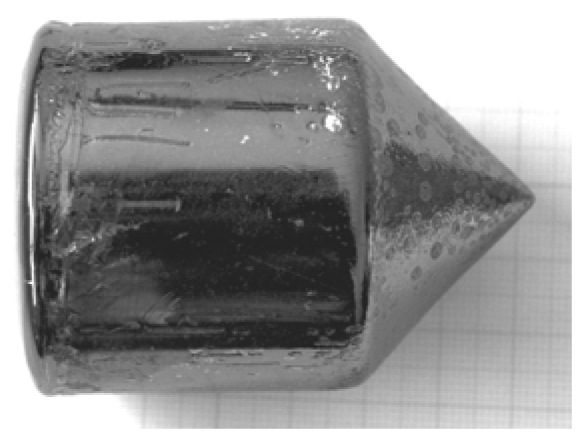
2-inch as-grown CdZnTe crystal.

The furnace has three independent heating zones. The thermal gradient at the melt/crystal interface is about 10 °C/cm. Before the growth, the pre-synthesized polycrystalline CdZnTe material is heat-treated in order to obtain a definite composition, than it is charged inside a quartz ampoule [44]. A boron oxide pellet is located above the material. At the beginning, the ampoule is kept in the upper part of the furnace. The temperature of the furnace is increased, so that at about 450 °C boron oxide starts softening. The furnace chamber is pressurized with inert gas at 6 – 10 atm. After the charge melting, the ampoule is lowered with a speed of 1 – 2 mm/h and the growth starts. No seeding procedure was ever employed. At the end of the growth, the crystal is removed from the ampoule by putting it into water. This causes the boron oxide to expand and break the ampoule walls. The eventual mass losses during the growth are determined by comparing the weight of the crystal and the weigh of the polycrystalline charge. Whenever growth procedure was correctly carried out, no mass losses were detected. In Figure 11 a 2-inch CdZnTe crystal is shown. The surface of the crystals is always shiny.

**Figure 12.**
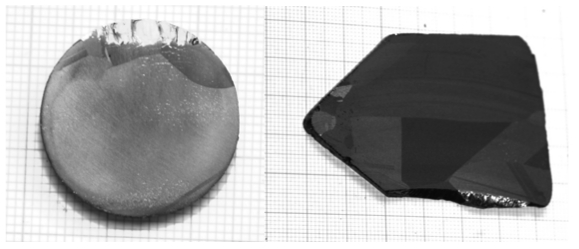
Large single grain wafers for 1-inch (left) and 2-inch (right) crystals.

The crystal is not completely single, however it is formed by large single grains, as shown in Figure 12.

**Figure 13.**
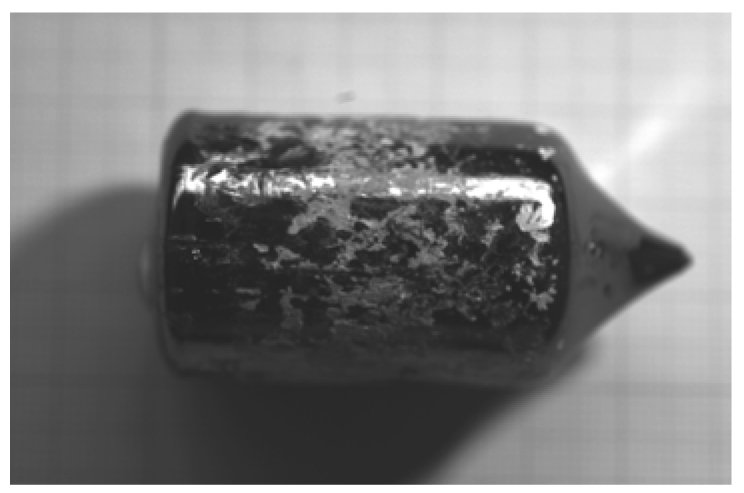
A CdZnTe crystal removed from the crucible: traces of boron oxide are evident on the crystal surface.

These characteristics suggest that the crystals grow without direct contact with the ampoule walls. This hypothesis was verified by carefully removing the crystal from the growth ampoule. The crystal was immersed into water just for a few seconds, so that boron oxide was only partially damped by water. After that, it was possible to see a boron oxide layer all along the crystal walls (see Figure 13). The thickness of the boron oxide layer is about 100 micron. The presence of this layer also justifies the presence of large single grains. In fact, generally, untreated quartz ampoules cause the formation of many spurious nuclei on the crystal walls.

The experiments show that during growth the whole crystal is surrounded by a liquid boron oxide layer, so that the crystal-crucible contact is prevented. However, the mechanism of formation of this layer must be further investigated. The melting point of boron oxide is much lower than that of CdZnTe, implying that boron oxide can flow along the crucible wall before the CdZnTe starts melting. However, once the charge is molten, the hydrostatic pressure is expected to push the boron oxide to the top of the ampoule. The reason for the formation of a stable liquid boron oxide layer along the crucible wall is the interaction between the quartz crucible and the boron oxide, resulting in the formation of a borosilicate glass.

**Figure 14.**
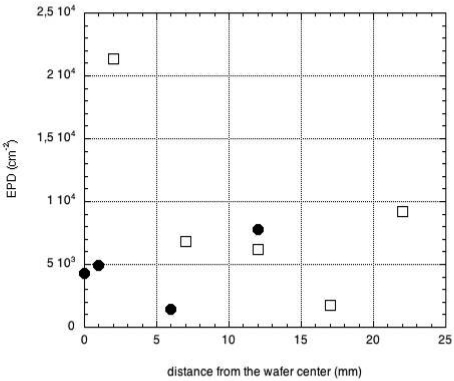
EPD in 1-inch (plain dots) and 2-inch (squares) CdZnTe crystals grown by the boron oxide vertical Bridgman technique.

In order to show that such interaction occurs, we have carried out electron dispersion x-ray analysis (EDX) on the crack surface of the crucible after growth. EDX spectrum shows the presence of boron peak close to the crucible edge on the crystal side, however, being boron a light element, the absolute determination of the concentration of boron leads to big errors. This is the reason why, in order to study the composition profile along the crucible section, we have considered the ratio between the silicon and the oxygen concentration. Far from the interface, the ratio is close to 0.5, as is due to pure quartz. At about 200 μm from the interface, the amount of silicon starts to decrease and reaches the minimum close to the interface. Borosilicate glass is formed due to the fact that the crucible is in contact with molten boron oxide at high temperature. Etch pit density (EPD) measurements were performed on (111) oriented surfaces of CdZnTe wafers using the Nagakawa etching in order to evaluate the dislocation density [45]. The pits appear randomly distributed. The value of the EPD along the radius of a wafer is shown in Figure 14, for 1-inch and 2-inch crystals. In the case of the 1-inch crystals, close to the border the EPD is maximum, while the minimum of EPD is at about half the wafer radius. This distribution is typical in compound semiconductors and is usually referred to as w-shaped distribution. However, it must be stressed that the EPD values in Figure 14 are always well below the 10^4^ cm^-2^ limit and, in particular, reach the minimum value of 1.5 × 10^3^ cm^-2^. The “w” distribution was not found for 2-inch crystals. However, also in this case, the EPD values are lower then 1×10^4^ cm^-2^ with the exception of few hot spots (see for example Figure 14) were 2 × 10^4^ cm^-2^ pits were counted. It must be stressed that these values are about one order of magnitude lower then the value typically reported for Bridgman grown crystals [35,36,46,47]. It is known that one of the reasons for the formation of dislocations is the mechanical stress induced by the crucible to the crystal. Thanks to the liquid boron oxide layer surrounding the crystal, this detrimental effect is avoided, thus the dislocation density is reduced.

### 4.2. Boron Oxide grown CdZnTe detectors

**Figure 15.**
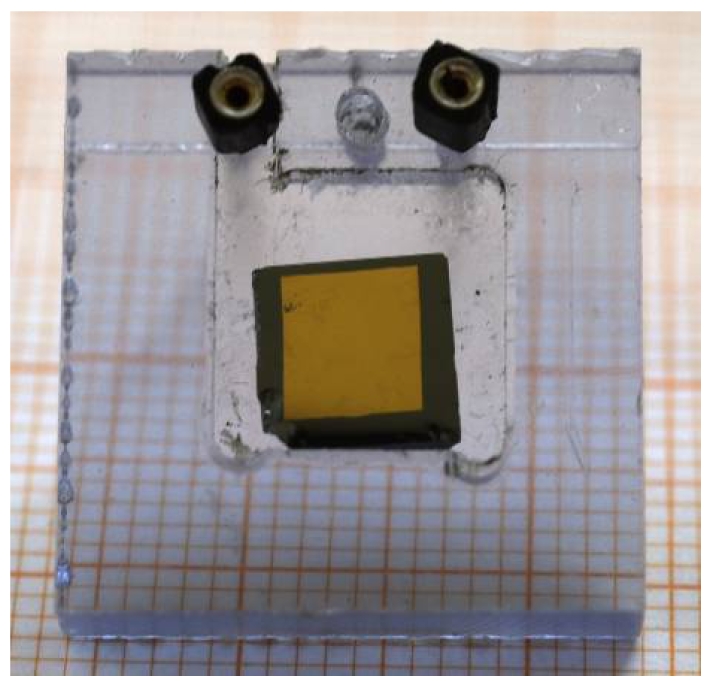
CdZnTe planar detector, equipped with gold electrodes, deposited by electro-less technique. The crystal is grown by the boron oxide vertical Bridgman technique.

Using CdZnTe ingots grown with the technique described above, different planar sensors have been prepared in order to investigate their performance as X-ray and gamma-ray detectors [48, 49]. The main characteristics of the detectors are: active surface of 4 × 4 mm^2^ and thickness of 1.10 mm. Detectors were irradiated with uncollimated radioactive sources (^241^Am, ^109^Cd) and the signals were readout by a conventional spectroscopic NIM electronic chain. Figure 15 shows the CdZnTe detector.

**Figure 16.**
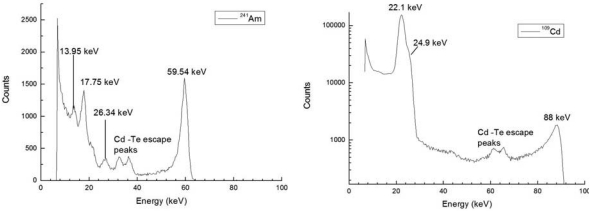
Spectra measured with a CdZnTe detector obtained by a crystal grown by the boron encapsulated vertical Bridgman technique, exposed to ^241^Am (left) and ^109^Cd sources (right).

The results of the characterization are given in Figure 16 (left: ^241^Am; right: ^109^Cd). The energy resolution (FWHM) at the 59.5 keV line of the ^241^Am source is 4.9%, and at the 88 keV line of the ^109^Cd source is 3.7% at room temperature. Moreover, many low energy lines of the two spectra are clearly resolved.

The measured mobility-lifetime product of the electrons presents a satisfactory value of 2 × 10^-3^ cm^2^/V (i.e. not very far from values of commercial CdZnTe), but the spectra shape demonstrates that there still are problems related to the interaction position with respect to the electrode: e.g. contact interface, non-uniform electric field and polarization effects.

## 5. Single Charge Carrier Sensing Detectors

As pointed out in the previous discussions, the poor hole transport properties of CdTe and CdZnTe materials are a critical issue in the development of X-ray and gamma ray detectors. Hole trapping reduces the charge collection efficiency of the detectors and produces an asymmetric long tail in the photopeaks of the measured spectra (hole tailing).

Several methods have been used in order to minimize this effect. Some techniques concern the particular irradiation configuration of the detectors. The *planar parallel field* (PPF) is the classical configuration used in overall planar detectors, in which the detectors are irradiated through the cathode, thus minimizing the hole trapping probability if the detector is thin. In an alternative configuration, denoted as *planar transverse field* (PTF) [50], the irradiation direction is orthogonal (transverse) to the electric field. In such configuration different detector thicknesses can be chosen, in order to fit the detection efficiency required, without modifying the inter-electrode distance and then the charge collection properties of the detectors. This technique is particularly useful to develop detectors with high detection efficiency in the gamma energy range.

Several techniques are used in the development of detectors based on the collection of the electrons (single charge carrier sensing detectors or unipolar detectors), which have better transport properties than that of the holes. Single charge carrier sensing techniques are widely employed for CdTe and CdZnTe detectors, by using both electronic methods (pulse rise time discrimination [51], bi-parametric analysis [52,53]) and by developing careful electrode design (Frisch-grid [54,55], pixels [56-58], coplanar grids [59], strips [60,61] multiple electrodes [62-65]).

*Risetime discrimination* (RTD) is based on the selection of preamplifier pulses with short rise time, which are principally due to electrons. This technique improves the energy resolution but leads to significantly reduced sensitivity since many counts may be rejected.

Another electronic method is based on the analysis of the correlations between the amplitude (pulse height) and the rise time of the signals (*bi-parametric analysis*, BP). The use of BP correction reduces the tailing and enhances both energy resolution and detection efficiency.

**Figure 17.**
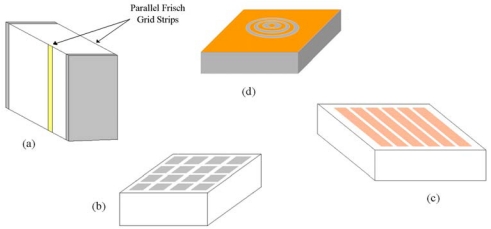
Single charge collection electrode configurations widely used in CdTe and CdZnTe detectors: (a) parallel strip Frisch grid design, (b) pixels, (c) strips and (d) multiple electrodes.

Alternatively, a careful electrode design gives to semiconductor detectors single charge collection properties (unipolar detectors). Figure 17 shows some unipolar electrode configurations widely used in CdTe and CdZnTe detectors.

The first single charge carrier sensing technique was implemented in gas detectors by Frisch [66] to overcome the problem of slow drift and loss of ions. A simple *semiconductor Frisch grid detector* can be built by using parallel metal strips on the opposite faces of the detector [yellow strips of Figure 17(a)]. *Pixels* and *strips* on the anode electrode of detectors [Figures 17(b) and (c)], mainly used for their position sensitive characteristics, are also characterized by unipolar properties. The *small central anode electrode* and the multiple ring electrodes on the anode surface of the detectors, as shown in Figure 17 (d), optimize the charge collection, minimizing the effect of the hole trapping on the measured spectra.

In general, the unipolar characteristics of these detector configurations are due to the particular shape of the weighting potential: it is low near the cathode and rises rapidly close to the anode. According to this behavior, the charge induced on the collecting electrode, proportional to the weighting potential, as stated by the Shockley–Ramo theorem, is mostly contributed from the drift of charge carriers close to the anode, i.e. the electrons. On the contrary, the linear shape of the weighting potential of a planar detector, makes the induced charge sensitive to both electrons and holes, as discussed above.

**Figure 18.**
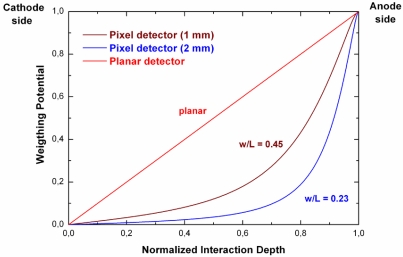
The weighting potential for pixel detectors and a planar detector as a function of the normalized interaction depth (interaction depth/detector thickness); the w/L parameter is the ratio between the pixel size and the detector thickness. The weighting potential and then the detector signals are more unipolar as the w/L ratio decreases, in agreement with the small pixel effect.

Figure 18 shows the weighting potential of a pixel detector, compared to a planar detector. It is possible to improve the unipolar properties of pixel detectors by reducing the w/L ratio (i.e. pixel size to detector thickness), according to the theory of the small pixel effect [56].

**Figure 19.**
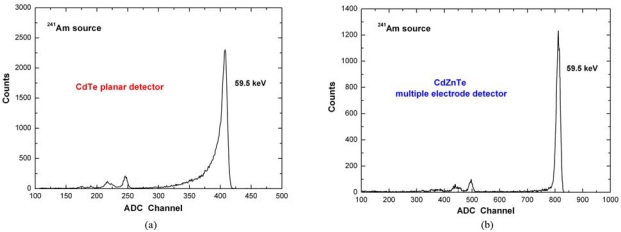
(a) The ^241^Am spectrum measured with the CdTe planar detector; it shows an energy resolution of 2.7 % FWHM at 59.5 keV (T = -20 °C) [67]. (b) The measured ^241^Am spectrum with the CdZnTe multiple electrode detector; it shows an energy resolution of 1.9 % FWHM at 59.5 keV (T = -10 °C) [64]. It is clearly visible the small tail of the 59.5 keV photopeak of the spectrum measured with the CdZnTe multiple electrode detector.

To better point out the spectroscopic improvements in CdTe/CdZnTe detectors equipped with unipolar electrode configurations, we reported in Figure 19 the ^241^Am spectra (59.5 keV) measured with a planar CdTe detector [67] and a CdZnTe multiple electrode detector with the same thickness [64]. The anode surface of the CdZnTe detector consists of a circular platinum electrode (the collecting electrode) surrounded by two ring electrodes and by one electrode that extends to the edge of the crystal [Figure 17(d)]. Both detectors together with the input FET of the electronics (DC-coupled) are cooled by using compact Peltier cells; further characteristics of both detectors have already been described in our previous works [64,67]. As shown in the Figure 19, the CdZnTe multiple electrode detector is characterized by low tailing and better energy resolution than that of the CdTe planar detector (CdTe: 2.7 % FWHM at 59.5 keV; CdZnTe: 1.9 % FWHM at 59.5 keV).

## 6. Astrophysical Applications

Recently both ESA and NASA have indicated in their guidelines for X-ray and gamma-ray astronomy in the next decade the development of new instrumentation working in the energy range from keV to the MeV region, where important scientific issues are still open. They have identified various priorities for the development of new instrumentation: (a) concentrating telescopes (e.g. multilayer mirrors) for hard X-rays (1 – 100 keV) and focusing instruments based on Laue lens operating from about 60 keV up the MeV. To exploit the performance of both multilayer hard X ray mirrors and Laue lenses, focal plane detectors with high efficiency and fine spectroscopy (e.g. a few % FWHM at 60 keV) and with a moderate spatial resolution (between 0.5 and 2 mm) are required. In particular for hard X-ray focal plane detectors and wide field instrumentation for gamma ray burst (GRB) monitoring, an energy threshold close to 1 keV will be very important allowing the measure of critical feature such as the Fe lines and a good overlapping with soft X-ray telescopes ranges. The high segmentation of the detectors and the required spectroscopic performance would also allow very sensitive measurements of the flux polarization level from high energy sources [68].

CdTe and CdZnTe are considered promising materials for the construction of position sensitive spectrometers with the required characteristics. These detectors have obtained a great attention from the scientific community interested in the X- and gamma-ray band applications and particularly in the realization spectroscopic imager for the universe exploration. As demonstrated by the several groups in the world working on this technology, many favorable conditions have contributed to confirm CdTe/CdZnTe as one of the most promising semiconductor for the realization of high performance spectrometers: good crystal growth technology with adequate homogeneity and purity; physical, chemical and thermo-mechanical stability, high mean atomic number and wide band-gap are all necessary conditions for obtaining high detection efficiency and functionality at non cryogenic temperature [69-71]. The capability of CdTe/CdZnTe detectors in astrophysical applications has already been demonstrated with the European astronomical satellite, INTEGRAL, (INTErnational Gamma-Ray Astrophysics Laboratory) and with the BAT instrument on board the NASA mission SWIFT [72]. INTEGRAL is an astronomical satellite for observing the gamma-ray sky. The satellite was launched on October 17, 2002 by a Russian PROTON launcher in a highly eccentric orbit with a revolution period around the Earth of three sideral days. It was chosen to minimize the background noise due to protons trapped in the radiation belt and to allow for long periods of unbroken observation. The satellite is still operative. The imager IBIS (Imager on Board the INTEGRAL Satellite) has been optimized for fine imaging and precise detection of radiation sources. The imager has a detector with a large number of pixels, all physically distinct. The detector uses two parallel planes of pixels located one on top of the other, thus allowing detection of both low and high energy photons. The top layer is made of 16,384 CdTe pixels and the lower layer is made of 4,096 CsI (Cesium-Iode) pixels. IBIS achieves an angular resolution of 12 arcmin over an energy range from 15 keV to 10 MeV [69].

There are several examples of focal plane detectors for the next generation of hard X-ray and gamma- ray telescopes based on both multilayer mirrors and Laue lens. These include: the French-Italian satellite mission Simbol-X (2012) [70], the European Gamma Ray Imager (GRI) mission (ESA Call for Cosmic Vision 2015-2025) [71] and the NASA NuSTAR mission [73]. Furthermore, the proposed detectors configuration can answer to the requirements of wide band and wide field new instrumentation such as it is required for GRB and/or all sky monitor in mission like LOBSTER [74] and EDGE [75] in which the extension both to low energies and to high energies can be particularly appealing for the scientific objectives.

The Simbol-X mission is basically designed to extend the X-ray focusing technique to much higher energies, up to ∼ 70 keV, i.e. well beyond the transition between thermal and non thermal emissions and will be a qualified instrument to elucidate the origin of the non thermal emission in accretion astrophysical sites, both compact and extended. For its baseline design, SIMBOL-X is built using a classical Wolter I optics focusing X-rays onto a focal plane detector system. The gain in maximum energy is achieved by having a long focal length, of 30 meters, i.e. four times that of XMM–Newton mirrors. Since this cannot fit in a single spacecraft, the mirror and detectors will be flown on two separate spacecraft in a formation flying configuration. The focal plane detector system will combine a silicon low energy detector, efficient up to ∼ 20 keV, on top of CdTe/CdZnTe high energy detector. They will be surrounded by an active CsI anticoincidence shield. The detector diameter will be 6 cm, slightly more than nominally required by the mirror Field of View. Both detectors are spectroscopic-imagers, and will have a pixel size of 500 mm maximum, which will allow a sufficient oversampling of the mirror point spread function (4.4 mm diameter HEW; HEW is the half energy width). Both detectors can also be read out at high speed, a fact which will be used to efficiently reduce the high energy background by using them in the anticoincidence scheme in addition to the CsI guard counter.

Lobster-ISS was an X-ray instrument initially proposed by an International collaboration (P.I. University of Leicester, UK) in response to the ESA call for two flexi-missions for the International Space Station (ISS). The project has been approved for an industrial study for accommodation on board the ISS Columbus External Payload Facility (CEPF). The main scientific objective of Lobster-ISS was the mapping of the X-ray sky in the 0.1 – 3.5 keV energy band with an angular resolution as low as 4 – 6 arcmin and a daily sensitivity of 2 × 10^-12^ erg cm^-2^ s^-1^. The main instrument is based on Micro-Channel Plate (MCP) optics in a Lobster-eye configuration and focal plane detectors based on special sensitive proportional counters. The Lobster project, in addition to the X-ray optics, included the development of a Gamma-Ray Burst Monitor (GRBM) with the minimum goal of identifying the GRBs detected by the X-ray telescope. The GRBM consists of 4 misaligned detection units, each one made of an array of Peltier-cooled CdZnTe, or SDC diodes coupled to CsI crystals, surmounted by a passive collimator which defines the field of view. The FOV of each unit is 35 × 55 degrees (FWHM), resulting in a total rectangular field of view of 35 degrees in the direction of the ISS motion and 240 degrees in the direction perpendicular to it.

In Italy there is a strong interest in astrophysical applications of CdTe/CdZnTe detectors and there are many activities actually in progress. These research lines are founded by Agenzia Spaziale Italiana (ASI), Istituto Nazionale di Astrofisica (INAF) and by the Ministry of Scientific Research (MIUR). In particular we discuss some activities on the development and characterization of CdZnTe detector prototypes.

### 6.1. GRI 3-D CdZnTe detector prototype

The GRI [71] mission was presented to the ESA Call for Cosmic Vision 2,015 – 2,025 and was not selected in the first round. However the mission is still under study in the framework of the ASI High Energy Study and will be re-submitted in a next round. GRI will for the first time ever make use of novel focusing optics to concentrate high-energy photons on a small focal spot. The energy coverage of 10 keV – 1.3 MeV will be achieved by combining a Laue lens with a single-reflection multilayer-coated mirror. The Laue crystal lens will be efficient throughout the 200 keV – 1.3 MeV energy range, while the multilayer mirror will cover the 10 keV – 250 keV hard X-ray band. The photons concentrated by the lens will be detected using a semiconductor stack detector optimized for high photo peak efficiency and low background noise throughout the relevant energy band. The GRI focal plane detector is based on a position sensitive spectrometer made of four stacked CdZnTe layers, surrounded by CdZnTe side walls. The stack will be composed of a 5 mm thick top layer, optimized for photoelectric absorption in the 10 – 250 keV band, and three bottom layers, each one 20 mm thick, to grant a total detection efficiency better than 75% for photons below 1 MeV. The detector stack is directly exposed to the source photons focused by the lens and mirror optics, while the side wall detectors are used to collect scattered photons from the primary beam in order to maximize the full energy absorption efficiency. The entire detector is surrounded by a segmented veto shield made of 44 BGO modules, which are read out through optically coupled new generation photomultiplier tubes. In the framework of a study for GRI mission, it has been designed a focal plane detector prototype.

**Figure 20.**
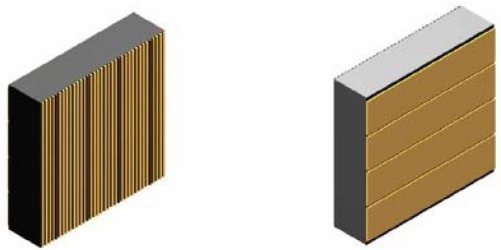
The basic CdZnTe sensitive units; (left) anode side with microstrip pattern: the brown strips are the collecting anodes (0.10 mm wide), while the others (yellow) are the drift strips (0.15 mm wide); (right) cathode side with the four horizontal strip for the reconstruction of the interaction position.

The driving idea for the realization of this prototype is the use of CdZnTe crystals in the so called PTF configuration to increase the photon absorption thickness up to 20 mm without increasing the charge collection distance (i.e. without severely degrading the spectroscopic performance). This sensor irradiation configuration can be coupled with the attractive possibility to use an anode made of an array of microstrips in drift configuration (a thin collecting anode strip surrounded by guard strips at decreasing bias) to improve the spectroscopic performance [57]. In order to add the 3D capability the cathode will be segmented in the direction orthogonal to the anode strips one (see Figure 20).

This crystal size of 10 × 10 × 2.5 mm^3^ has been chosen because these dimensions are quite standard for all the CdZnTe companies on the market, minimizing the procurement costs and lead time. In order to have square pixels (2.5 × 2.5 mm^2^) on the photon entrance surface of each CdZnTe crystal and taking into account the requirement to have the collecting and guard strips width less than 1/10 of the crystal thickness [76], for an effective spectroscopic improvement, it is possible to divide the anode in four sets of microstrips with the structure shown in Figure 20, being the pitch between each collecting strip 2.5 mm. On the opposite CdZnTe side, the cathode will be segmented in four 2.5 mm wide strips. At the end the chosen electrode configuration will define on each CdZnTe crystals a perfectly cubic voxel (Volume pixel) of 2.5 × 2.5 × 2.5 mm^3^. This prototype will be operative in 10 – 1,000 keV energy band [77].

### 6.2. The POLCA(POLarisation with Cdznte Array) detector

Polarimetry has been recognized as a very important observational parameter for high energy astrophysics (> 100 keV), as confirmed also by very recent and important results obtained from SPI/INTEGRAL data on the polarization of the Crab pulsar [78]. Therefore the capability to perform an accurate polarimetric measurement of high energy cosmic sources should really be included in proposals for future space missions but it has also become very appealing in the last three years in the development of a new telescope mission concept based on Laue focusing techniques.

The principle of operation as a polarimeter of a X and γ-ray detector is based on the ability to detect Compton scattered events. In fact the impinging photon polarisation status introduces a typical asymmetry of the double events (photons that first undergo a Compton interaction and are then absorbed through a second interaction with the material) distribution [78]. Therefore each segmented detector can be used in principle as a scatter polarimeter, but in particular CdZnTe/CdTe pixel detectors are very suitable for this type of measurements at high energy (> 100 keV) due to the fine segmentation achievable [79]. These detectors, due to their intrinsic material properties and geometric shape promise efficient polarimetric measurements between 100 keV and 1 MeV.

**Figure 21.**
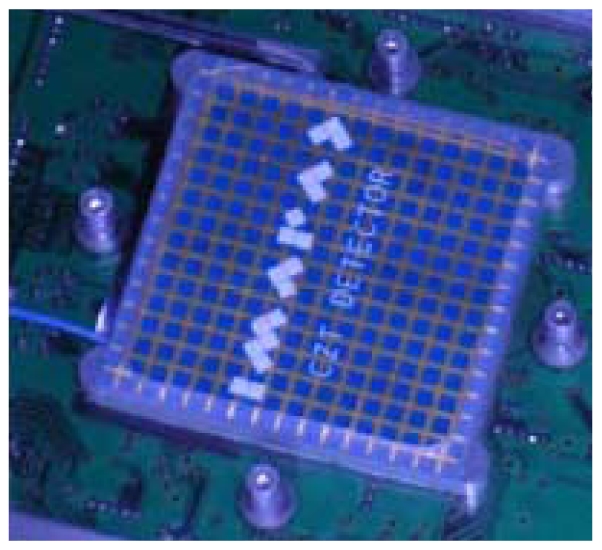
The POLCA CdZnTe detector.

In order to assess the polarimetric performance achievable with CdTe/CdZnTe pixel detectors, it has been developed a prototype with the proper coincidence logic to perform experiments with linear polarised beam: the POLCA (POLarisation with Cdznte Array) detector comprises a CdZnTe pixel spectrometer from IMARAD (Israel) and an analog front-end electronics. The detector is made of two adjacent CdZnTe crystals (5 mm thick) for a total area of 4 × 4 cm^2^. The anode side is segmented in a 16 × 16 array (256 pixels) of 2.5 mm pitch, while the cathode is a mono-electrode (Figure 21). The detector is connected through 3 high density connectors with a custom designed board containing 8 ASICs (eV Products) with 16 independent channels each.

The main results of the POLCA experiment obtained by means of several measurement sessions performed at the ESRF (Grenoble) have been recently published [80].

### 6.3. CdZnTe pixel detectors for hard X-ray telescopes

**Figure 22.**
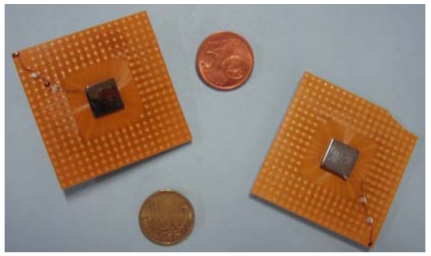
CdZnTe pixel detectors (cathode side view). Both detectors are characterized by an anode array of 256 pixels (pitch 500 μm).

The good room temperature performance and the high spatial resolution of pixellated CdZnTe detectors make them very attractive as focal plane detectors for hard X-ray telescopes. Italian research activities on the development of focal plane detector prototypes for balloon-borne X-ray telescopes operating in the 20 – 70 keV energy range, were carried out [58,81-83]. In the framework of these activities CdZnTe pixel detectors have been designed and developed. The detectors are based on CdZnTe crystals (10 × 10 × 1 mm^3^; 10 × 10 × 2 mm^3^); the anode surface consists of a 256 pixel array surrounded by a guard ring, which was extended to the edge of the crystal. The array is characterized by a fine pixel pitch of 500 μm in both directions: 450 μm is the pixel size with a gap of 50 μm. The cathode is a planar electrode covering the entire detector surface. Both anode and cathode are made by platinum sputtered on the crystal surface in order to realize ohmic contacts. The detectors are mounted by using a “flip-chip bumping” electrical interface on a custom board, as shown in Figure 22.

The detectors are characterized by good energy resolution at room temperature (5.8 % FWHM at 59.5 keV for the 1 mm thick detector; 5.5 % FWHM at 59.5 keV for the 2 mm thick detector) and low tailing in the measured spectra, confirming the single charge carrier sensing properties of the CdZnTe detectors equipped with a pixellated anode layout.

## 7. Medical Applications

Over the last decade, increasing effort has been devoted to the development of devices for medical applications based on CdTe and CdZnTe detectors [84-86]. CdTe and CdZnTe detectors as X-ray and gamma ray spectrometers are very competitive with traditional systems based on solid scintillators, silicon and germanium detectors. Advantages and disadvantages of CdTe and CdZnTe detectors in medical systems are reported and discussed, pointing out both international and Italian research activities.

### 7.1. Nuclear Medicine

In nuclear medicine a gamma-ray emitting radiotracer is usually injected intravenously in the body and its distribution is imaged by using dedicated systems. Medical imaging systems include mainly nuclear cameras (Anger type cameras) and positron emission tomography systems (PET). Both systems are energy dispersive: nuclear cameras operate mainly at 140 keV (gamma emission of ^99m^Tc), while PET systems make use of coincident detection of 511 keV positron-annihilation gamma rays (positron emission of ^11^C, ^13^N, ^15^O).

**Figure 23.**
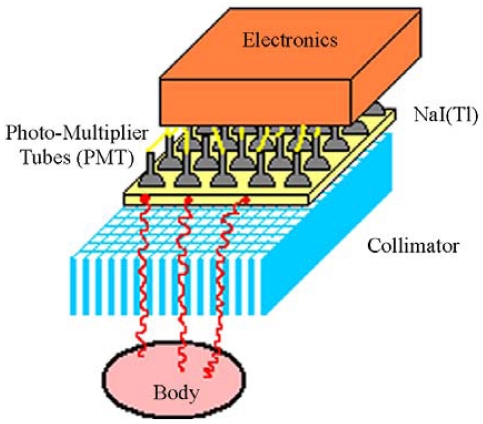
The typical elements of a gamma camera. Gamma rays emitted by the medical target pass through the collimator and are detected within the camera, which generates data related to the location of scintillations in the crystal as well as to the energy of the gamma rays. The scintillation light resulting from the interaction of a gamma ray in the NaI(Tl) crystal is viewed and localised by photomultiplier tubes (PMT).

The most common gamma camera is based on an original design developed by Anger in the 1950's [87]. Figure 23 shows the basic elements of a gamma camera. The heart of the device consists of a large-area of NaI(Tl) scintillator viewed by an array of position sensitive photomultiplier tubes (PMT).

In a gamma camera both the position and the energy information of the recorded photons are important. The energy information is used to discriminate Compton scattered rays and thus to improve the image characteristics (contrast, signal to noise ratio).

The intrinsic spatial resolution of current gamma cameras is typically 3 – 4 mm (FWHM) and the energy resolution is rather poor (11% FWHM at 140 keV) [85]; in particular the degraded energy resolution at lower energies (16% FWHM at 60 keV [85]) is a critical issue for both the intrinsic spatial resolution and the intrinsic efficiency. Interests in detection systems with sub millimeter intrinsic spatial resolution and better energy resolution, stimulated the development of new gamma cameras based on CdTe and CdZnTe detectors [88-90]. CdTe and CdZnTe detectors show better energy resolution than NaI(Tl) detectors; unipolar electrode geometries and electronic corrections were used to overcome the incomplete charge collection due to the hole trapping. Moreover, it is possible to segment these detectors into pixel arrays with very fine pitch allowing high spatial resolution. Consequently, several gamma camera prototypes, based on arrays of single CdTe/CdZnTe detectors or of pixellated detectors have been developed in the last decade [88,91-94]. Table 2 lists the principal characteristics of gamma cameras based on NaI(Tl) (Anger type camera) and CdTe/CdZnTe detectors. Recent gamma camera detector prototypes based on pixellated CdZnTe detectors showed excellent energy resolution (3% FWHM at 140 keV [94]) and good intrinsic spatial resolution < 2.5 mm (FWHM) [94].

**Figure 24.**
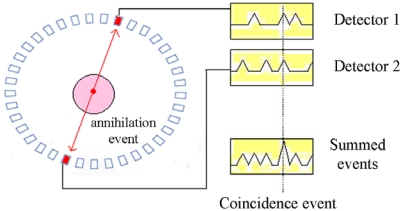
The typical PET system geometry. The medical object being scanned is usually surrounded by rings of detectors. Each detector records single gamma-ray events and generates a timed pulse associated to each incident photon. These pulses are then combined in coincidence circuitry in order to select the paired gamma rays from a single annihilation.

A PET system utilizes coincidence detection of the 511 keV photons from electron-positron annihilation. Since the paired gamma rays from the annihilation of the positron are anti-parallel, the detection of the gamma rays determines a line of response along which the annihilation took place. Typically, the PET systems are based on solid scintillators (BGO, LSO, etc) and photo detectors (PMTs, photo diodes), designed on a ring layout (Figure 24).

These systems showed high efficiency, good position resolution of the order of few millimetres, excellent timing performance and low cost. However, growing interest for imaging of small animals and for molecular imaging required small PET systems with higher spatial resolution than the conventional systems. CdTe and CdZnTe detectors show great potentialities for small PET systems [95-99]: the well known good spatial and good energy resolution and especially the three-dimensional (3D) pixellization for a 3D localization of photon interaction which is very important to minimize parallax errors. The poor timing performance of the CdTe and CdZnTe detectors, due to the low mobility of the charge carriers, are the major drawback for PET systems. However, for small PET systems, the small size of the subjects and the restrictions on dose administered make the timing properties of CdZnTe*/*CdTe less critical.

Recently, segmented CdZnTe detectors with bi-parametric corrections and PTF irradiation showed both good energy resolution and good timing properties (coincidence time of 2.6 ns FWHM at 511 keV [98]).Table 3 illustrates the principal characteristics of some PET detector prototypes.

**Figure 25.**
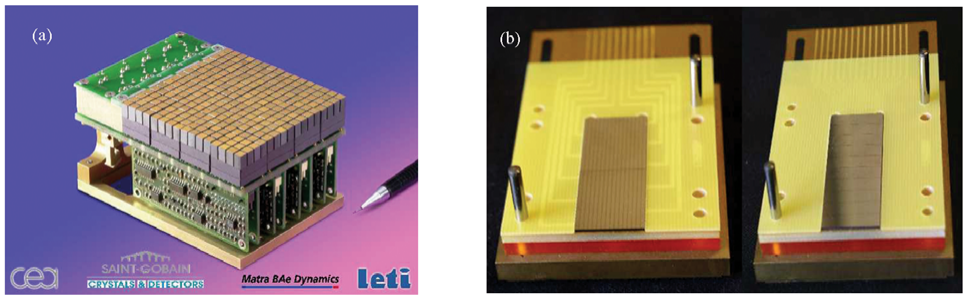
(a) CdZnTe detector prototype for gamma cameras [93]; it consists of 256 single CdZnTe detectors (4 × 4 × 6 mm^3^) assembled to realize a small imager. (b) Anode (left) and cathode (right) side view of a PET detector prototype based on two CdZnTe strip detectors irradiated in PTF configuration [98]. The 511 keV photons were absorbed in 40 mm thick CdZnTe detector. These figures are kindly provided by L. Verger (LETI-CEA-MINATEC Recherche Technologique, CEA, Grenoble, France).

Figure 25 shows two CdZnTe detector prototypes for gamma camera (Figure 25(a) [93]) and PET (Figure 25(b) [98]).

### 7.2. Mammography

**Figure 26.**
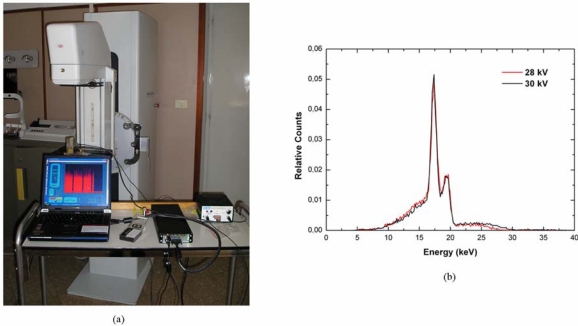
(a) Experimental set-up of direct measurements of mammographic spectra with a portable device based on a CdTe detector. (b) Mammographic X-ray spectra (Mo target) measured with a portable device, based on a CdTe detector, under clinical conditions [67]. The mammographic tube settings were: 60 mAs, 28 kV and 30 kV.

CdTe and CdZnTe detectors are also suitable for the development of portable systems for mammographic X-ray spectroscopy [67,100-102]. A typical mammographic unit, based on a X-ray tube with Mo target [103], produces X-ray spectra characterized by a continuous distribution (1 – 40 keV) with discrete sharp lines superimposed (Mo fluorescent lines: K_α_ = 17.44 keV and K_β_ = 19.63 keV). Measurements of the X-ray spectra are very important in quality assurances and quality controls of mammographic systems, especially for radiation protection calculation, patient dosimetry and optimization of imaging properties. Several portable prototypes, based on solid state detectors, have been proposed for direct measurements of mammographic spectra [104-106]. CdTe and CdZnTe detectors compete with Ge and Si detectors. Despite the excellent energy resolution of Si and Ge detectors, several distortions due to their low detection efficiency and secondary X-ray escape are visible in measured X-ray spectra [105,106]; moreover, the use of large cryogenics systems in Si and Ge detectors is a critical issue for X-ray measurements under clinical conditions. Thin CdTe/CdZnTe detectors (1 mm thick) give good efficiency, good energy resolution and low tailing in the measured spectra. In this context, Italian research groups are involved in the development of portable systems, based on CdTe and CdZnTe detectors [67,102,107,108]. A portable system [67], based on a 1 mm thick CdTe detector, is characterized by an energy resolution of 5% FWHM at 22.1 keV, no escape events and low tailing in the measured spectra. Figure 26(a) illustrates the experimental set-up of mammographic measurements with a portable system, based on a CdTe detector, under clinical conditions; Figure 26(b) shows two spectra of the mammographic unit (Mo target) measured with the CdTe detector.

## 8. Discussion

In this paper a review of the development of CdTe and CdZnTe detectors is given with a particular emphasis on activities carried out in Italy. Among the various applications in this continuously growing field, we point out examples in medical and astrophysical fields. The research and development activities, currently in progress, involve a quite large research group geographically well distributed and representing different research institutions, aim mainly at two goals: (i) the development of complete and working detector prototypes for medical and astrophysical applications, also within the framework of international projects, and (ii) the growth of spectrometer-grade CdTe and CdZnTe crystals in Italian laboratories. In particular this second research line, that recently gives quite good results, will allow to the Italian scientific community a better autonomy in the procurement of the crystals, at present solely of foreign production.

## Figures and Tables

**Table 1.** Physical properties of the principal compound semiconductors at T = 25 °C.

**Material**	**Si**	**Ge**	**GaAs**	**CdTe**	**Cd_0.9_Zn_0.1_Te**	**HgI_2_**	**TlBr**
**Crystal structure**	Cubic	Cubic	Cubic (ZB)	Cubic (ZB)	Cubic (ZB)	Tetragonal	Cubic (CsCl)
Growth method*	C	C	CVD	THM	HPB, THM	VAM	BM
**Atomic number**	14	32	31, 33	48, 52	48, 30, 52	80, 53	81, 35
**Density (g/cm^3^)**	2.33	5.33	5.32	6.20	5.78	6.4	7.56
**Band gap (eV)**	1.12	0.67	1.43	1.44	1.57	2.13	2.68
**Pair creation energy (eV)**	3.62	2.96	4.2	4.43	4.6	4.2	6.5
**Resistivity (Ω cm)**	10^4^	50	10^7^	10^9^	10^10^	10^13^	10^12^
**μ_e_τ_e_ (cm^2^/V)**	> 1	> 1	10^-5^	10^-3^	10^-3^ - 10^-2^	10^-4^	10^-5^
**μ_h_τ_h_ (cm^2^/V)**	∼ 1	> 1	10^-6^	10^-4^	10^-5^	10^-5^	10^-6^

* The more common growth methods: C = Czochralski, CVD = chemical vapor deposition, THM = traveler heater method, BM = Bridgman method, HPB = high-pressure Bridgman and VAM = vertical ampoule method

**Table 2.** Characteristics of gamma cameras based on NaI(Tl) and CdTe/CdZnTe detectors.

**Gamma cameras**	**Characteristics**		

	**Detector properties**	**Energy resolution at 140 keV (%)**	**Intrinsic spatial resolution (mm)**
Anger type camera [94]	NaI(Tl) 9.5 mm thick	11	3.5
NUCAM [91]	40 × 32 array of CdTe detectors; pixel size (4 × 4 mm^2^)	5	*
Digirad 2020tc Imager™ [88]	64 CdZnTe modules (25 × 25 × 5 mm^3^); each module 8 × 8 array of pixel	4	*
NUCAM3 [92]	528 CdZnTe pixel detectors; (8.5 × 8.5 × 5 mm^3^) 4 × 4 array of pixel	4.5	2.1
PEGASE [93]	Groups of 16 single CdZnTe detectors (4 × 4 × 6 mm^3^)	4.7	*

* not reported

**Table 3.** Characteristics of PET systems based on LSO and CdZnTe detectors.

**PET Detectors**	**Characteristics**	

	Time resolution (FWHM)	Energy resolution at 511 keV (%)
LSO; 0.975 × 0.975 × 12.5 mm^3^ [98]	3 ns	15
CdZnTe; 16 × 5 × 10 mm^3^.Anode segmented [96]	10 ns	2
CdZnTe; 16 × 20 × 0.9mm^3^ Anode and cathode segmented [98]	2.6 ns	2
